# Engagement and Influence of Food-Related Videos on Social Media Within the Dietary Realities of Young Adults in Jamaica and the United Kingdom: Qualitative Cross-National Study

**DOI:** 10.2196/89720

**Published:** 2026-10-19

**Authors:** Shaniek Parks, Charlotte Albury, Jessica Renzella, Asha Kaur, Suzanne Soares-Wynter, Mike Rayner, Peter Scarborough

**Affiliations:** 1 Nuffield Department of Population Health, University of Oxford, Old Road CampusOxford, England, OX1 3JA, United Kingdom; 2 Nuffield Department of Primary Care Health Sciences, University of Oxford, Oxford, England, United Kingdom; 3 Caribbean Institute for Health Research, University of the West Indies, Kingston, Jamaica

**Keywords:** social media, food-related videos, young adults, Jamaica, United Kingdom, digital food environments, qualitative research, focus groups, health behaviors, diet, algorithms, nutrition

## Abstract

**Background:**

The rise of video-based social media platforms and the shift toward a video-first content strategy by social media companies have altered how people consume and engage with food-related content. However, little is known about how young adults experience, interpret, and are influenced by food-related videos on social media across different national and cultural contexts.

**Objective:**

This paper explores how young adults in Jamaica and the United Kingdom experience, interpret, and are influenced by food-related videos on social media.

**Methods:**

Twelve focus groups (online and in-person, 6 in Jamaica and 6 in the United Kingdom) were conducted between August and November 2024. Sixty participants attended the focus groups; after excluding data from 7 individuals suspected of misrepresenting their eligibility, the final dataset included 53 participants aged 18‐25 years. Discussions explored their experiences with food-related videos. Transcripts were analyzed using reflexive thematic analysis as the foundation, complemented by framework analysis, and the constant comparison method to facilitate cross-national and cross-cultural comparison.

**Results:**

Our analysis generated four themes: (1) young adults balance algorithmic influence with personal curation; (2) young adults engage with food-related videos for relaxation, amusement, and learning; (3) young adults evaluate food-related content through inquiry and strategy—using quick credibility checks such as comparing sources, reviewing comments, assessing transparency, and considering cultural context before deciding whether to trust or ignore content; and (4) food choices form at the intersection of online and offline worlds. A unified high-order theme, “a journey of influence,” captures how young adults move from exposure to evaluation and filter digital experiences through offline realities, leading to change or continuity in dietary attitudes and behaviors. Although this journey was consistent in Jamaica and UK focus groups, the ways young adults interpreted food-related content varied culturally.

**Conclusions:**

This cross-national qualitative study showcases how and why young adults engage with food-related videos on social media, and how these interactions shape their attitudes and behaviors toward food and nutrition. Our conceptual map visualizes the influence of food-related videos as a dynamic process unfolding across digital and offline environments, identifying 3 decision points for intervention: exposure, evaluation, and contextualization. These findings highlight opportunities to design youth-responsive digital nutrition interventions that recognize young adults’ agency and the sociocultural and material realities that shape their food choices and eating habits.

## Introduction

The rise of video-based social media platforms such as TikTok (ByteDance) and YouTube (Google LLC), and the prioritization of a video-first content strategy by other social media platforms [1] and marketing professionals [2], have transformed how people consume and engage with food-related content. Visually appealing videos are now dominant on social media, capturing users’ attention and shaping their decisions [3,4]. Worldwide, more than 5 billion social media user accounts exist [5], and an estimated 5.5 billion people use the internet [6], indicating that video-based social media content reaches a large segment of the global population.

There are over 1 billion food-related videos on TikTok [7], highlighting the huge presence of food content on social media. Evidence shows that food-related videos can contribute to body image concerns [8,9] as well as influence purchase intentions [10], food preferences [11], and dietary behaviors [12,13]. In this paper, “food-related videos” refers to social media videos in which food, eating, diet, or nutrition is a central focus. This includes user-generated, influencer, and commercial videos about preparing, purchasing, presenting, promoting, consuming, or reviewing food, as well as nutrition advice and cultural food practices.

A growing body of research has examined the effects of food videos on children [14-16] and teenagers [12,13], but less is known about how young adults experience, interpret, and are influenced by these videos on social media. This age group (18-25) represents a key life stage in which many rely on digital platforms for health information [17,18] but have poorer dietary health compared to other adult age groups [19,20]. Given the prevalence of obesity and overweight, which remain a global health priority, understanding the digital factors influencing young people’s dietary behaviors is vital for developing targeted healthy eating programs and obesity prevention initiatives.

While food-related videos on social media transcend national boundaries, existing research has focused on single-country contexts. This limits our understanding of how cultural, social, and economic factors shape engagement with similar content in different countries, and to what extent context-specific public health responses are required. Scholars have therefore identified a need for more comparative cross-national [21] and cross-cultural studies [22,23].

We selected Jamaica and the United Kingdom as study locations to explore how different contexts shape young adults’ engagement with food-related videos on social media. While both countries have high internet penetration, 96% in the United Kingdom [24] and 83% in Jamaica [25], and high rates of overweight and obesity among adults [26,27], they differ in terms of health policy maturity, sociocultural context, food environments, and economic structures. The United Kingdom is a high-income country with advanced digital infrastructure and well-established public health policies targeting diet-related noncommunicable diseases (NCDs), including restrictions on online promotions of foods high in fat, sugar, and salt. Jamaica, by comparison, is an upper-middle-income country at a pivotal point in its policy response to the obesity epidemic. Several of its strategic plans outlining its response to growing levels of obesity and NCDs expired in 2018 and 2020 and are due for an update. This creates an opportunity to implement new, targeted strategies for the next phase of Jamaica’s policy response to obesity and NCD prevention.

This paper addresses the age-group and cross-national comparison gaps identified in the literature by exploring young people’s engagement with food-related videos in the United Kingdom and Jamaica. The research question guiding this investigation is: What are the experiences of young adults in Jamaica and the United Kingdom with food-related videos on social media?

To answer the research question, we pursued 2 objectives, which were the following: (1) explore how and why young people engage with food-related videos on social media and (2) explore the influences of food-related videos on young people’s attitudes and behaviors related to food and nutrition.

## Methods

### Ethical Considerations

Ethics approvals for this study were obtained from the University of Oxford’s Medical Sciences Interdivisional Research Ethics Committee (R82824/RE001) and the University of the West Indies’ Mona Campus Ethics Research Committee (CREC-MN.0226, 2023/2024). This study’s protocol is registered on the Open Science Framework [28]. Study participants received gift vouchers valued at £30 (United Kingdom) and J $6000 (Jamaica). At the time of writing, the currency exchange rate of US $1=£0.75 and US $1=J $159.

### Study Design

We adopted an idealist ontology to explore how young people understand their realities and experiences with food-related videos. Our epistemological position combined interpretivism and pragmatism: interpretivism informed our recognition that knowledge is co-constructed with participants, while pragmatism supported using these insights to inform intervention and policy development. Guided by this stance, we used a descriptive qualitative methodology that emphasized naturalistic inquiry and allowed the selective use of frameworks to support interpretation [29].

We conducted semistructured, moderator-led focus groups using a topic guide with open-ended questions, while allowing participants to raise issues relevant to them. To support accessibility and equitable participation [30], focus groups were held online and in-person in each country.

SP developed the study design and initial focus group topic guide (Table S1 in Multimedia Appendix 1), drawing on discussions with coauthors and findings from a recent systematic review of the behavioral and cognitive changes in young adults after exposure to digital food communication [31]. SP also convened consultations with Young People Advisory Groups (YPAGs) in Jamaica and the United Kingdom who advised on the study’s focus, reviewed the topic guide, and recommended focus group duration and group size. After these consultations, SP reviewed existing literature to better define the study focus and identify the research gap related to food-related videos, resulting in an improved study design and refined topic guide. Our guide was piloted in July 2024, and revisions were made based on the online and in-person pilot sessions and early focus groups.

### Participants and Recruitment

Eligible participants were aged 18‐25 years, had lived in Jamaica or the United Kingdom for at least five years, and used video-based social media platforms to watch food-related videos weekly.

We used purposive sampling, recruiting through youth and community organizations, social media, and snowballing, as participants were encouraged to invite members of their network to register for subsequent focus groups. Interested individuals were directed to a Qualtrics page containing an introductory video, the Participant Information Sheet, and the Informed Consent form. After consenting, participants completed a screening questionnaire (Material S1 in Multimedia Appendix 1). Those who met the inclusion criteria were invited to complete a registration survey that collected demographics, video-viewing habits, and availability (Material S1 in Multimedia Appendix 1). These data supported scheduling and were used to create a case classification sheet summarizing demographic characteristics.

### Data Collection

One researcher (SP) conducted 6 focus groups per country, guided by the finding by Guest et al [32] that 90% of themes are apparent within 3 to 6 focus groups. Our decision was reinforced by the paper by Malterud et al [33] on information power. Each 90-minute focus group included 4 to 7 participants to facilitate discussion and engagement [34,35]. Online focus groups were conducted on Microsoft Teams, and in-person focus groups were held at community centers, churches, and seminar or conference rooms. Participants were invited to focus groups that matched their preferences, availability, and location listed in their registration forms. Focus groups were not stratified by age, gender, or other demographic characteristics. All focus groups were audio- and video-recorded, and transcribed verbatim. In Jamaica, a research assistant observed one online and one in-person focus group and took field notes from both sessions. Focus group discussions centered on participants’ experiences with food-related videos. They also described surrounding platform features and practices when explaining how they encountered, engaged with, and evaluated these videos, including social media feeds, comment sections, advertisements, and external searches. All participants were assigned pseudonyms as unique identifiers, and data were stored on password-protected university drives. We did not conduct repeat focus groups; transcripts were not returned to participants, and no one chose to withdraw from this study.

### Impostor Participants

We suspected that 7 participants in the first 2 online UK focus groups had misrepresented their eligibility for this study. Here, the word “impostors” refers to individuals suspected of misrepresenting their eligibility for this study by providing inaccurate information to meet the eligibility criteria. Based on existing literature, we noted that these participants fulfilled several indicators of impostors [36,37], including providing vague answers, distracted participation, and a preoccupation with the timeline of payment for participation. Other indicators included difficulty coming on or staying on camera, answers showing unfamiliarity with the UK context when mentioning UK supermarkets or food companies, and grounding their responses in references unrelated to the United Kingdom. Technical indicators, including emails with timestamps associated with time zones outside the United Kingdom, provided additional reasons to question their eligibility. Our concerns were therefore based on a combination of behavioral, contextual, recruitment, and technical inconsistencies. Importantly, neither interest in the financial incentive nor a lack of willingness or inability to use a camera was considered a valid reason for exclusion on its own.

We considered several options to improve the trustworthiness of the data, including repeating the affected focus groups, excluding all data from those focus groups, retaining all the data, or removing only the contributions of suspected impostor participants. A key consideration was that we did not want to remove the contributions of genuine participants who gave their time and energy in service of this research. We therefore used a two-pronged approach to preserve data trustworthiness.

1. For focus groups with suspected impostor participants, audio and video recordings were reviewed. Following discussions among coauthors and an exploration of published guidance [36,37], we decided to exclude the 7 suspected impostor participants’ data from transcripts before analysis. The contributions of participants whose eligibility was not in question were retained. The exclusions were intended to protect the integrity of our study by ensuring that the analyzed data came from eligible participants.
2. Following these incidents, we paused the remaining online UK focus groups and initiated a process whereby we asked individuals previously scheduled for focus groups whether they would be open to a brief introductory one-on-one meeting with SP, allowing her to evaluate their eligibility, the consistency of the information they had provided during recruitment, and to identify combinations of indicators previously observed among suspected impostor participants [36]. During the meetings, SP asked individuals to speak briefly about their lives in the United Kingdom, and invited them to participate using their camera. If, at the end of the meeting, SP suspected that someone was an impostor or could not verify their eligibility, she thanked them for their interest in this study and informed them that we would not proceed with them as a participant. This screening process was used before the remaining online UK focus groups. The numbers invited to, attending, and excluded after these introductory meetings were not recorded.

### Researcher Reflexivity

We acknowledge that data collection and analysis are a cocreation of the focus group participants and SP, a female doctoral student of Jamaican heritage enrolled at the University of Oxford. SP was the lead moderator for all 12 focus groups conducted in Jamaica and the United Kingdom and subsequently led the data analysis. In preparation for this study, she received training in qualitative data collection and analysis, and the use of NVivo (Lumivero). SP had no prior relationship with the majority of the participants. However, she previously met 6 UK participants and 4 Jamaican participants through university and religious group affiliations. SP’s Jamaican heritage may have supported rapport with Jamaican participants and her interpretation of culturally specific accounts. Her Oxford affiliation and prior familiarity with some participants may also have shaped how participants perceived her and what they felt comfortable sharing.

SP entered this study with an awareness, informed by existing literature and consultations with YPAGs in Jamaica and the United Kingdom, that food-related social media content could influence young adults’ food-related attitudes and behaviors. She also recognized that her Jamaican heritage and position as a researcher living and studying in the United Kingdom could shape her interactions with participants and interpretation of similarities and differences between the countries. SP used field notes and memos maintained in NVivo during coding to reflect on data collection, coding, and theme development. Her interpretations and positionality were discussed with the coauthors who served as “critical friends”.

### Data Analysis

Cross-national and cross-cultural comparisons between Jamaica and the United Kingdom were key components of our analysis. We selected reflexive thematic analysis [38] as the overarching basis for our analysis because of its flexibility. Within our reflexive thematic analysis, we used the framework analysis method [39] to organize codes into categories and develop an analytical framework that supported systematic comparison across focus groups. We used the constant comparison method [40] throughout the data analysis process, starting at the coding stage, to compare participants’ experiences within and across countries in order to refine codes and categories, and it played a key role when developing and refining themes. Our analysis was consistent with the six stages of reflexive thematic analysis developed by Braun and Clarke [38]; full details are provided in Table S2 in Multimedia Appendix 1. Participant quotations are presented verbatim; some Jamaican participants spoke in Patois, which was kept to preserve authenticity.

SP led the analysis, including familiarization with the data, inductive coding of all 12 focus-group transcripts, development and refinement of the analytical framework, and generation of the initial themes. One focus group from each country was initially coded and discussed with CA, AK, and PS. The developing codebook, analytical framework, and themes were subsequently discussed with the research team. Coauthors acted as “critical friends” by questioning SP’s interpretations, considering alternative explanations, and supporting reflection on how her positionality shaped the analysis. Our work is reported in keeping with COREQ (Consolidated Criteria for Reporting Qualitative Research) in Checklist 1.

## Results

### Demographic Information

We conducted 12 focus groups, 6 per country, between August and November 2024. Sixty young adults participated in the focus groups, comprising 29 participants from the United Kingdom and 31 from Jamaica. Data from 7 UK individuals suspected of misrepresenting their eligibility were excluded before analysis. The demographic data and transcripts analyzed therefore represented 53 participants, 22 from the United Kingdom and 31 from Jamaica.

Table 1 summarizes the self-reported demographics of the 53 participants in aggregate and by country. Almost half were aged 18‐20, one-quarter were 21‐23, and just under one-third were 24‐25. Most identified as female, with fewer identifying as male or nonbinary. Over half lived in urban areas, and just over one-third lived in rural areas. Most were full-time students, and almost half were employed in some capacity.

**Table 1.** Demographic characteristics of focus group participants (N=53).

Characteristic and category	Overall (N=53), n (%)	United Kingdom (n=22), n (%)	Jamaica (n=31), n (%)
Age group (years)			
18‐20	24 (45.3)	6 (27.3)	18 (58.1)
21‐23	13 (24.5)	7 (31.8)	6 (19.4)
24‐25	16 (30.2)	9 (40.9)	7 (22.6)
Gender			
Female	34 (64.2)	14 (63.6)	20 (64.5)
Male	18 (34.0)	7 (31.8)	11 (35.5)
Nonbinary	1 (1.9)	1 (4.5)	0 (0.0)
Rural-urban classification			
Urban	34 (64.2)	21 (95.5)	13 (41.9)
Rural	19 (35.8)	1 (4.5)	18 (58.1)
Employment status			
Student - full-time	31 (58.5)	11 (50.0)	20 (64.5)
Employed - full-time	13 (24.5)	5 (22.7)	8 (25.8)
Employed - part-time	9 (17)	5 (22.7)	4 (12.9)
Student - part-time	6 (11.3)	2 (9.1)	4 (12.9)
Self-employed	3 (5.7)	2 (9.1)	1 (3.2)
Other - out of work and looking for work; out of work but not currently looking for work	2 (3.8)	2 (9.1)	0 (0.0)

Table 2 displays participants’ self-reported engagement with different types of food-related videos on social media. Recipe videos were the most frequently viewed, with nearly half of the participants watching them multiple times per day. Taste-test and reaction videos, and health-related or diet videos also showed high daily engagement. “What’s in my fridge?” videos were the least watched, with almost half of the participants reporting that they seldom or never watched them.

**Table 2.** Focus group participants’ engagement with different types of food-related videos. Food brand/food product videos are promotional or informational videos created by brands showcasing specific food items. Grocery haul videos involve individuals sharing and discussing items purchased during a shopping trip. Health-related/diet videos include content focused on nutrition advice, diet trends, or healthy eating tips. Private chef videos typically feature chefs preparing meals and sharing their experiences and skills. Recipe videos demonstrate how to prepare meals or snacks. Restaurant vlogs showcase experiences of dining out, exploring menus, and reviewing restaurants. Taste test and reaction videos involve individuals sampling new, unusual, or trending foods and sharing their reactions. “What’s in my fridge?” videos feature content creators revealing and discussing the food items they store in their refrigerators at home. What-I-eat-in-a-day videos document a creator’s meals during a typical day.

Video types	Multiple times a day (%)	Once a day (%)	Every few days (%)	Weekly (%)	Monthly (%)	Seldom (%)	Never (%)
Food brand/food product videos	18.9	17	24.5	11.3	11.3	13.2	3.8
Grocery haul videos	17	13.2	13.2	13.2	11.3	20.8	11.3
Health-related/diet videos	26.4	15.1	20.8	18.9	7.5	7.5	3.8
Private chef videos	20.8	20.8	15.1	13.2	11.3	11.3	7.5
Recipe videos	45.3	9.4	20.8	13.2	3.8	1.9	5.7
Restaurant Vlogs	24.5	11.3	28.3	7.5	9.4	11.3	7.5
Taste tests and reaction videos	28.3	20.8	20.8	11.3	5.7	11.3	1.9
What’s in my fridge videos	11.3	7.5	13.2	9.4	11.3	30.2	17
What-I-Eat-in-A-Day	15.1	18.9	37.7	5.7	9.4	7.5	5.7
Other food influencer videos	34	11.3	20.8	15.1	5.7	5.7	7.5

### Themes

#### Overview

During analysis, we generated four themes: (1) young adults balance algorithmic influence with personal curation; (2) young adults engage with food-related videos for relaxation, amusement, and learning; (3) young adults evaluate food-related content through inquiry and strategy; and (4) food choices form at the intersection of online and offline worlds. The 4 themes are unified by 1 high-order theme: “a journey of influence,” which tells the story of how online exposure, engagement, and evaluation processes are filtered through offline realities with the potential to shape young adults’ dietary behaviors and attitudes. We present each theme with illustrative anonymized quotes, labeled by focus group, participant ID, gender, age, employment status, and urban-rural classification.

#### Theme 1: Young Adults Balance Algorithmic Influence With Personal Curation

Young adults’ encounters with food-related videos illustrate an ongoing negotiation between the influence of algorithms and personal choice. Many participants expressed a sense of being watched by algorithms but also of trying to gain mastery over their feeds through curation, showing how digital environments can simultaneously restrict and empower. Here, we use the term “personal curation” as defined in digital media literature to refer to users’ selective engagement with the content available to them, including decisions about what to view, follow, ignore, or remove [41]. In the following paragraphs, we explore this theme through participants’ accounts of how they feel algorithms shape the food-related videos that they see and their efforts to resist or redirect its influence.

Algorithms are the foundation for exposure on social media feeds and For You Pages (FYP), determining what content is shown to platform users. An FYP is a section of the Instagram and TikTok apps, curated by algorithms, that shows users a personalized stream of videos based on assumed interests and engagement patterns. In contrast, a social media feed displays content from the accounts a person follows, and it may also show advertisements and content the algorithm thinks the user may find interesting.

Participants in both countries recognized the powerful role of algorithms in selectively exposing them to food-related videos. What they saw also varied by platform. For example, YouTube was described as useful for long-form and short-form recipe videos, while TikTok was associated with food trends and targeted marketing. The algorithm’s invisible curation eventually led participants’ social media feeds to display content that appealed to them and encouraged engagement. Some participants reported that algorithms used unintentional actions, such as liking or mindlessly watching a video, to push similar food-related content at them. One UK participant explained his experience with content he had never consciously engaged with, which always appeared on his feed.

*It landed in my Instagram reel algorithms, but then something piqued my interest around it. So I guess I spent some time engaging with it or looking at it, and then the algorithms learned, so they keep on feeding me more. But I think it was never like a really conscious decision. It just kind of landed there.*

UK#4 [UK16, male, 25, student, urban]

Other participants in both countries sought to exercise agency over the food-related videos they encountered by actively curating their feeds to attract preferred content. This included following accounts for food-related content and influencers of interest, and repeatedly liking food videos that they wanted to see more of. These actions represented small but intentional efforts to influence what they were exposed to and consumed, as illustrated by the quote below.

*So when I'm curating like my For You Page and the content that I consume, I ensure that the cooking videos and food videos and pages that I follow are tailored to be more healthy.*

JAM#2 [JM5, female, 19, student, employed, urban]

Young adults in both countries also exercised agency to reduce unwanted exposure and filter out distractions. They used social media platform settings to block specific creators, avoid videos not related to their interests, unfollow accounts, or select “not interested” to stop certain types of unwanted food-related videos from appearing in their social media feeds and FYP. For example, one participant described deliberately using these platform tools to avoid content he found repulsive.

*I really hate [seeing] a brisket being cooked. Basically, like, oh, there’s this one guy… He will buy a brisket… He'll put this open it out, put it in his sink. And just, I don't know, he just throws me off so bad. I have to go into the share part and put, “not interested” and I had to block a couple people as well. It’s gotta work*.

UK#5 [UK23, male, 18, student, urban]

Another participant went further, viewing resistance as a way to recalibrate his digital environment and start afresh. He described deleting his social media account and creating a new, alternate profile to reset his feeds, regain control, and improve his experience. A “Finsta,” or “fake Instagram,” refers to a secondary Instagram account used to share private or unfiltered content with a small group of close friends.

*I was sick of it. So then I had this alter account and I deleted the original account. So, it started off kind of like Finsta, then became my main account. And I really enjoy it much more. It really shows you that after resetting the algorithm, you can have a completely different and better experience.*

UK#3 [UK15, male, 24, employed, urban]

Still, others felt that algorithms undermined their control, repeatedly displaying unwanted content despite their efforts to personalize their feeds. This created a lingering sense of frustration and powerlessness.

*We kind of don't have control over the types of food videos we see, in terms of, yes, we can organise our algorithms and stuff, but at the same time…For example, mukbang videos, I may not necessarily want to see that, but the algorithm might still be pushing that on me.*

JAM#2 [JM5, female, 19, student, employed, urban]

These reflections show participants in both countries oscillating between awareness and frustration, recognizing the algorithm’s logic while feeling unable to fully control it. Notably, some differences were apparent in how participants made sense of algorithms across the two countries. In the Jamaican focus groups, participants expressed less cynicism about the algorithm; instead, they viewed it as something they simply had to work with. In contrast, some UK participants approached the algorithm with mocking detachment and suspicion, describing it as “a spy” that anticipates desires before they are consciously formed. This sense of being watched produced unease, as these participants mocked the algorithm’s intrusiveness while continuing to engage with its suggestions. The exchange below captures a conversation among 3 participants in a UK focus group, in which they discuss algorithms mockingly and suspiciously.

***UK20:*** *It comes up. Because you like it consistently, because the little spy [is] in your TikTok. He then puts them on your For You page.*

***UK23:*** *The algorithm. It’s the algorithm.*

***UK24:*** *A little spy, yeah?*

***UK20:*** *Yeah. It is a spy. Yeah, algorithms, that’s what it’s called, not spy on TikTok.*

UK#5 [UK20, female, 18, student, employed, urban; UK23, male, 18, student, urban; UK24, female, 24, out of work and looking for work, urban]

Participants in both countries noted that algorithms adjusted to their circumstances, often showing gym content after they joined a gym, student meal ideas at university, or travel-related foods when planning trips. In the same sessions, participants expressed both frustration and appreciation, criticizing algorithms for feeling intrusive yet valuing the tailored content shaped by their interests and search history. The quote below illustrates this tension.

*For me, especially on TikTok, I don't know if [it is] because my algorithm knows that I like hip hop and food. I'm seeing specifically a channel called Ayo Cheetah. Basically, it’s them rapping and showing how to cook. So I've seen that video a lot, which is fun.*

JAM#5 [JM21, male, 25, student, employed, rural]

This theme highlights the negotiation between young adults and algorithms that control the food-related videos they see on their social media feeds and FYPs. Participants in both countries were aware of algorithms’ roles and simultaneously expressed frustration and an uneasy appreciation of their value in exposure to food-related videos.

#### Theme 2: Young Adults Engage With Food-Related Videos for Relaxation, Amusement, and Learning

While participants expressed strong feelings about the influence of algorithms, their continued engagement with food-related videos was grounded in the clear benefits they offered. Enjoyment, inspiration, and emotional comfort motivated young adults to curate their feeds or deliberately search for food-related content that they wanted to watch rather than disengage.

Enjoyment was a key reason why young adults engaged with food-related videos, with participants describing sensory pleasure and appreciation for creativity in meal preparation and presentation. In both countries, participants described strong responses to ASMR (autonomous sensory meridian response) videos that focus on specific eating or cooking sounds designed to create a soothing sensory response. Many participants viewed these as a source of calm, pleasure, and relaxation. Mukbangs, a form of ASMR in which individuals consume large amounts of food, were popular for their sensory appeal. One UK participant vividly captured this attraction.

*Mukbangs. I love mukbangs… That’s where people like eat the food and don't talk. Love it, I do. I love the sauces they put on, the clanking of the mouth. I do. I love all lots of and I like when they make like at home meals, like ready meals at home. Yeah*.

UK#5 [UK20, female, 18, student, employed, urban]

While enjoyment of food-related videos was shared in both countries, its links to creativity and aesthetics were more prominent in Jamaican participants’ accounts. They often expressed appreciation for content that highlighted the artistry and effort put into meal preparation, with enjoyment arising from both how the food was made and presented, as well as from the food itself. Presentation, dress, and attention to detail were seen as reflections of pride, discipline, and respect for food, as shown in the quote below.

*The ASMR is kinda satisfying. She [the influencer] nah mek no chop, chop, chop loud noises. Nothing is wrong with that, but she isn’t making any loud noises. She ah tek her time and she ah go into detail wah she ago use the seasoning, everything step-by-step and it’s just pleasing to watch. And she always dress in fancy clothes… she just dress like she ah cook fi eat dinner*.

JAM#5 [JM23, female, 20, student, employed, urban]

For some, the enjoyment of ASMR food videos lies in the shared experience, such as watching them while eating their own meals or with another family member. One participant discussed using food-related videos to bond with a neurodivergent sibling.

*So, me and my younger brother, we don't really have much things in common and he is neurodivergent… now like whenever YouTube uploads a [calm cooking] video, we now wait for each other and we watch it together so now we have that little bonding moment, which is really nice.*

UK#1 [UK2, female, 25, employed, rural]

Curiosity also motivated participants to deliberately seek out ideas, techniques, and trends to try, making food-related videos a practical source of inspiration. Some young adults engaged intentionally with food-related videos out of curiosity about new food trends. They searched for examples to better understand how others adopt emerging food practices, as described by one UK participant below:

*So, I started recently…started just like drinking chia water like everyday, but then I would search on TikTok [for] ‘chia water, just to see like how people were making it nice and stuff and people would often just like have lemon or something and they would talk about their health benefits.*

UK#3 [UK15, male, 24, employed, urban]

Others engaged because food-related videos functioned as a convenient source of practical food knowledge. For some, this meant watching recipe videos for ideas to incorporate into weekly cooking and meal preparation, as the participant below explained.

*I like watching people cook and give their recipes, mainly like salad recipes or food I can make in the week, meal prepping for the week. That’s my main go-to cooking videos.*

JAM#3 [JM12, female, 18, student, urban]

Participants also described TikTok as their primary platform for finding quick recipes, preparation techniques, and nutritional ideas. UK participants noted that the platform had increasingly replaced search engines such as Google in their everyday search for information.

***UK23:*** *Anything you want to know, just search into the TikTok search box.*

***UK20:*** *I don't go Google no more. I just search: How do I do this?*

UK#5 [UK23, male, 18, student, urban; UK20, female, 18, student, employed, urban]

Several participants in both countries reported deliberately engaging with food-related videos to learn about new cultures. This highlights that learning through food was another motivation for engagement, going beyond the amusement, curiosity, or recipe inspiration previously discussed. For these young adults, food videos provided a window into global diversity and cultural expression, as the participant below explained.

*For me, it’s just a cultural exposure. I am very intrigued by learning [about] different cultures*.

JAM#5 [JM25, male, 20, student, urban]

This theme demonstrates that young adults choose to engage with food-related videos because they offer information, enjoyment, and relaxation. These motivations were shared in Jamaica and the United Kingdom, suggesting shared motivations for engagement despite differing cultural contexts.

#### Theme 3: Young Adults Evaluate Food-Related Content Through Inquiry and Strategy

##### Overview

Interpretation and evaluation of food-related content play important roles in the meaning-making process. Young adults described a layered evaluative process through which they scrutinized food-related content before deciding how to respond. This vetting process involved detecting “red flags,” assessing tone and transparency, interrogating credibility and sponsorship, cross-checking advice, and scanning comment sections for signs of inauthenticity. In both countries, participants gravitated toward content that signaled honesty or transparency, although cultural context shaped what “realness” looked like. Through this process, they determined what to trust, how, when, and to what extent they consumed different forms of food-related content. Our findings are presented through two related aspects of evaluation: assessing information, credibility, and commercial intent; and judging cultural authenticity and relevance.

##### Evaluating Information, Credibility, and Commercial Intent

Young adults shared that their limited understanding of nutrition shaped how they interpreted and questioned food-related content. Some participants expressed uncertainty about what “eating healthy” even meant, as evidenced in the quote below.

*I remember thinking, “Oh, I need to eat healthy.” And then I was like, “What does it mean to eat healthy?”. And then, you know, we've all been taught very basically, like, oh, eating healthy is a balanced diet, but what does that actually mean?*

UK#1 [UK2, female, 25, employed, rural]

This uncertainty exposes a wider problem, namely the lack of a baseline understanding of how to adopt a healthy diet and evaluate questionable dietary information. Participants noted that online content often replaced facts with personal opinions and unverified advice. This foundational knowledge gap made participants more susceptible to misleading content that blurred the line between evidence and personal preference. As a result, expertise alone no longer guaranteed trustworthiness, as anyone could claim to be an expert online. Participants in both countries described videos that appeared educational but contradicted established guidance on balanced diets and recommended food group intake, fueling doubt about whom or what to believe. One UK participant captured this confusion when comparing conflicting dietary advice promoted by influencers:

*So, there is one person on Instagram who… I think they're pretty much on a carnivore diet and they talk about … “Don't eat fruit, don't eat fruits. You don't really need veg. You just need meat, like organ meat and like eggs and stuff like that” … There’s another that’s called like the superhuman diet, where this person— they eat like a lot of meat. They eat a lot of eggs again, but they do eat fruit this time. And it’s like if you were in a vulnerable position, you would believe this. But then you've got two people, one is like: Oh, don't eat fruit but eat carnivore and this other one who’s basically carnivore but eat fruit. It’s like, how do you know what you're supposed to be listening to? And like there, there’s no one consensus.*

UK#1 [UK2, female, 25, employed, rural]

Participants’ awareness of their own vulnerability prompted caution. Caution involved staying alert to potentially misleading or exaggerated claims and avoiding automatic acceptance of advice that they could not verify. Although they recognized that food and health information should be checked against reliable sources, some felt unwilling or unable to take on this burden. As the UK participant below explained, caution meant recognizing the limits of her own expertise and the difficulty of distinguishing facts. By being guarded, young adults positioned themselves as self-protective learners within an environment they knew to be unreliable.

*I think I am guarded when it comes to like health videos more generally, and how food influences your health, because I think that there is a lot of mis/ disinformation about that online and especially about…Yeah. Internal biological workings, and especially because I'm not an expert in that, I maybe would find it hard to like fact check what is actually science-based and what isn't*.

UK#3 [UK14, female, 23, employed, urban]

Faced with contradictions about what constitutes healthy eating and an awareness of their own vulnerability, participants in both countries interrogated the credibility of food-related videos, relying on cues such as tone, consistency, and transparency to decide what to believe. This was a major topic, given that content creators dominate the social media space. As a starting point, participants assessed the messenger’s credibility. They expressed a baseline trust in qualified experts such as doctors and dietitians, but this trust was conditional. Here, one Jamaican participant stated that they trusted certified doctors who evaluated and explained videos.

*There are facts that people look at. There are also doctors, certified doctors, who I would say I trust, who will look at the videos and explain what’s happening.*

JAM#3 [JM12, female, 18, student, urban]

The moment an expert appeared commercially motivated, whether through sponsorship, brand partnerships, or selling their own products, their credibility diminished. This reveals how young adults evaluate information based on the messenger’s expertise and perceived integrity, recognizing that financial incentives can distort authenticity. Below, one UK participant discusses this type of interrogation.

*There was this influencer I used to follow who was a dietician, like accredited, and she would always react to videos where people were making health claims and call out for misinformation and then bring up a study. She … came out with her own protein powder line and everyone was like: this feels like a complete flipping the script. And like…her protein powder could have been perfect but if you go from calling out everyone to doing it yourself. I just think she lost a lot of credibility from that. And then I personally just stopped watching… But… I don't necessarily believe you as much now because you're buying into the culture that you were denouncing before*.

UK#3 [UK15, male, 24, employed, urban]

In both countries, concerns about credibility and sponsorship extended beyond influencers to the wider advertising ecosystem. Participants were skeptical of brand partnerships and product placements, often questioning whether the content was genuine or merely designed to persuade audiences to purchase. This awareness of marketing tactics shaped how they read influencer posts and formal advertisements, highlighting digital literacy around persuasion. For many participants, advertising signaled inauthenticity, as illustrated in the quote below.

*Even [with] Jamaican food content persons, I see a lot of Juici Patties ads. They’re like, “This is the best fried chicken ever,” and I know it’s not the best fried chicken ever because I tried it before. So, yeah, no…don’t trust them.*

JAM#4 [JM17, female, 21, student, rural]

This questioning of credibility and authenticity extended to how influencers presented themselves and their content. Participants reflected on the aesthetic choices in food-related videos, such as lighting, editing, and perfection, that shaped their perceptions of reality and trust. These visual cues symbolized the gap between online performance and daily life, leading some to question whether influencers were sharing authentic experiences or crafting aspirational ones to conceal manipulation. The quote below captures this sentiment.

*I do actually question whether…if it’s on TikTok, if it’s being produced for views or likes or clicks. If they edit out like the mistakes or like they make sure the surface is all clean and everything. So, it seems quite perfect which isn't reality. So, when I'm cooking it doesn't look like that and so I do approach with a little bit more scrutiny… It kind of feels like I'm being marketed towards.*

UK#3 [UK12, female, 23, student, urban]

This suspicion of aesthetically pleasing content seems to contradict participants’ reflections about choosing to interact with food-related content because it appeared polished (theme 2). This suggests both an appreciation of and frustration toward food-related content. This contradiction also highlights the strong appeal of such content: even when young adults recognized the “fakeness” or high production value, they still found themselves drawn in. Some participants noted that simply knowing something is an advertisement did not weaken its pull. The quote below captures one UK participant, a pescatarian who dislikes advertisements, describing seeing a McDonald’s McRib promotion on social media and admitting to feeling tempted.

*I’ve seen the McRib; makes you want to eat meat again. I can’t lie. Because that McRib from Mackees does make me wanna eat meat.*

UK#5 [UK20, female, 18, student, employed, urban]

Although this participant confessed that she did not actually purchase or eat meat as a result of the advert, the quote captures the tension between recognizing an advertisement, questioning its credibility, and still feeling persuaded by it. However, some participants did report succumbing to the allure, leading to food purchases and/or consumption. This suggests that awareness of persuasive intent does not always diminish the appeal of polished or repeatedly shown food-related videos.

Several participants in both countries sought to verify claims by relying on external sources or the comment sections of videos to gauge consensus and spot inconsistencies before deciding whether to believe them. As one UK participant below explained, fact-checking has shifted from traditional search engines to AI tools that provide quick clarification. However, this highlights a paradox that young people’s strategies for resisting misleading information on social media often rely on digital systems that can be sources of misinformation and bias.

*I mean it was mainly the comment section, but now it’s I've replaced Google with AI. So instead of scrolling through like all the Google search results, I just ask ChatGPT*.

UK#6 [UK28, female, 18, student, urban]

Even as participants used comment sections for fact-checking, they remained aware of their limitations. Several described recognizing patterns of inauthentic engagement, such as automated or overly positive comments that signaled the presence of bots or coordinated promotion. This awareness reflects another layer of digital literacy: participants assessed content and interrogated the social signals surrounding it, recognizing that manipulation could extend beyond the video itself.

*Some of the videos, I feel like they have bots in the comments. And they just automatically agree with whatever the video is saying and just try to make everyone think that it’s good.*

JAM#4 [JM18, female, 19, student, rural]

The comments section was also used to find signs of sponsorship or an undisclosed commercial relationship when assessing whether content was trustworthy. Below, one Jamaican participant explained the cues and their meaning.

*Usually, once it’s an ad, the company is tagged or they say, “This video is sponsored.” But a lot of the time, they try to play it off that the video is not sponsored, yet the company is in the comment section, because I read comments thoroughly.*

JAM#6 [JM30, female, 25, student, rural]

Young adults gravitated toward content that signaled honesty or transparency. Participants described developing a sense of what felt trustworthy, identifying cues such as unsponsored reviews, consistent messaging, or creators who appeared motivated by genuine interest rather than profit. As one UK participant below explained, explicitly stating that content was not sponsored served as a “green flag,” which is a reassuring marker of authenticity in an environment saturated with hidden marketing.

*I think like a green flag for me is often when they explicitly say that they're not sponsored. They're just like reviewing something because that’s the type of content they make or that they're trying to sort of maybe look at the more like science-y side of things. But yeah, I think like that, like explicitly saying that you're not, that it’s not an ad or you're not sponsored or anything. That is a green flag that I look for*.

UK#4 [UK16, male, 25, student, urban]

Conversely, participants in both countries recognized when food videos crossed the line from informative to harmful, instilling fear or guilt. In response, they disengaged and avoided content to protect themselves. Below, one Jamaican participant explains that fear-based content can be emotionally taxing and unrealistic, leading them to avoid viewing these videos.

*Sometimes it scares you, because they're like: “what if I eat this and that happen to me.” But I tend to try and avoid those now because we gotta eat*.

JAM#3 [JM12, female, 18, student, urban]

##### Evaluating Cultural Authenticity and Relevance

Cultural context also shaped what “realness” meant in both countries. Participants in the United Kingdom expressed a wide range of cultural identities, describing their food culture as not a single national “UK culture” but as Pakistani or Irish, for example. Religious cultures also played an important role in the United Kingdom. For instance, Muslim participants described how halal rules and Ramadan practices shaped their interpretation of food-related content. These cultural frames shaped how they linked food to heritage, community, and belonging, and they used them to judge online representations of their culture as genuine or appropriated.

One UK participant, as illustrated below, described how cultural belonging and historical awareness informed these judgments, revealing that credibility in digital food spaces is connected with politics and lived experience.

*Food is tied to a lot of people’s heritage. So, for example, if I saw a Korean man making Pakistani food, I would be distrustful because I'm like, well, why are you making it? You know, there’s plenty of Pakistani creators out there… And it’s very judgmental. But I think that just comes with a history of, you know, a lot of people of colour —they are constantly going through microaggressions and racism, but then what we always tend to find is people who commit those microaggressions are always so happy to take our food and the yummy bits of our culture, but not the rest. So, I do get a little bit distrustful there.*

UK#1 [UK2, female, 25, employed, rural]

Jamaican participants often described a shared attachment to national cuisine and, in some accounts, a reluctance to embrace unfamiliar dishes. Compared with UK participants, who described diverse cultural identities, Jamaican participants’ accounts more often drew on shared understandings of Jamaican culture, cuisine, and what it means to be Jamaican. This pattern of loyalty to Jamaican foods reflects how cultural belonging shaped digital engagement. The participant below articulates this attachment.

*Well, we are in the Caribbean, so more likely we are going to see Jamaican or Caribbean food, and for us Jamaicans, we are a bit picky. So, the food we're going to like the best or mostly… [is] Jamaican food. We're not a society that actually likes to dibble-dabble all around… We like our culture…We don't like to try new foods.*

JAM#6 [JM30, female, 25, student, rural]

A related but distinct pattern involved how Jamaican participants judged authenticity. While cultural loyalty reflected identity and belonging, authenticity focused on trust. Participants often approached unfamiliar dishes with caution, distancing themselves or showing disinterest, and dismissed non-Jamaican foods as less relevant to their daily lives. Foods rooted in Jamaican cultural knowledge felt trustworthy, whereas those that diverged from familiar preparation methods or flavor profiles did not inspire the same confidence. This sits interestingly alongside theme 2, where participants enjoyed watching food videos to learn about new cultures, yet still used Jamaican cuisine as the benchmark for judging other foods.

*I mean, I'm not judging, you know, different cultures they prepare their food in the way that they know how to prepare it. But you know, certain things I'm not going to try. You know, I would prefer eating the way that I know in our culture, I know how to prepare that certain type of food. So, it’s all good.*

JAM#1 [JM30, female, 25, student, rural]

In the United Kingdom, authenticity was sometimes seen through appreciation and adaptation rather than solely through cultural loyalty. This view reflects a more individualized approach to food culture, where meaning is formed through participation and appreciation instead of just heritage, as shown below.

*I live on the island of Ireland, and [in] Northern Ireland [the food] and it’s not the most flavoursome and so I enjoy going outside. For example, looking more like Asian [foods] specifically is what I would generally look for. I enjoy sort of the spice and how the spices all come together and make a very tasty dish. So, I don't know if I'm doing it very well, but I'm following the recipes to the best of my ability, so it’s tasty for me.*

UK#1 [UK1, male, 20, student, employed, urban]

Theme 3 reveals that participants evaluate food-related videos through a dynamic process of sense-making, where skepticism and openness coexist as they interpret what feels real, relevant, familiar, and trustworthy. They are deliberate and adaptive, using strategies to fact-check, filter, and emotionally reframe what they encounter to maintain a sense of control. It is noteworthy that the cultural contexts differed between the two countries, with UK participants describing diverse cultural identities instead of a single “UK culture,” which shaped how they interpreted food videos. In contrast, Jamaican participants often drew on a shared national identity surrounding food when interacting with food-related videos.

### Theme 4: Food Choices Form at the Intersection of Online and Offline Worlds

Food-related videos inspired reflection and action among young adults, but their influence was not straightforward. Digital content introduced ideas about what to eat, how to cook, and what a “healthy” lifestyle should look like, but its impact was filtered through offline realities, such as income, access to ingredients, time, and social context. In Jamaica and the United Kingdom, participants balanced aspiration with realistic expectations, drawing inspiration from food videos while negotiating what was feasible in their everyday lives. This theme explores how digital influence intersects with offline constraints that anchor young adults’ choices.

Participants in both countries shared how food videos influenced their attitudes and behaviors. In some cases, the impact was immediate, prompting spontaneous decisions to try a new food or recipe. In others, influence was gradual, shaping outlooks and small behavioral shifts over time, from experimenting with ingredients to reconsidering entire diets. As one UK participant explained, watching a familiar creator discuss veganism introduced a new perspective on food, which prompted a lasting dietary change.

*A few years ago, I think it was like six years now. I watched a video about like a YouTuber that I liked, who was vegan. And it was talking about veganism and then like a couple of months later, that’s what inspired me to become vegan. And so, I feel like that was a direct link and like, it wasn't the main reason, but obviously it was. I watched that video and I'd never really considered it before until I saw that video*.

UK#6 [UK26, nonbinary, 22, employed, urban]

Feelings of disgust also led to changes in eating habits. Jamaican participants were extremely expressive about this, linking visual discomfort toward foods considered raw or uncooked by cultural standards to real-world dietary decisions. For example, the participant below temporarily stopped eating beef after watching a social media video.

*I remember this lady making beef and it was so raw that any time I look at beef, I was going to vomit, so I stopped eating beef for a whole month*.

JAM#3 [JM14, female, 18, student, urban]

However, the extent to which food videos translated into real dietary change depended on participants’ offline realities, particularly their capacity and readiness to act on what they saw. In both countries, healthy eating was viewed through the lens of life stage and affordability. Young adults described health-oriented lifestyles as desirable but deferred them as something to grow into when they are older, financially stable, or more settled.

Participants spoke extensively about risk-taking and its acceptability at this stage of their lives. Many described being a young adult as a period in which smoking, eating unhealthily, and “taking chances” felt permissible, in ways they believed would not be justifiable when older. A sense of physical invulnerability reinforced this, as several participants noted that they had not yet felt the effects of unhealthy eating, which made these behaviors seem less consequential. At this stage of life, experimentation and indulgence were accepted parts of youth, while “eating healthy” was reserved for a more mature future self. This framing positioned food-related videos on healthy eating as aspirational instead of actionable.

*We are experimenting and in our stage [of life] most persons, you could tell them that going down that path is wrong. Let me experience it for myself. So, you could do so much campaigning; it doesn't stop me from going out there drinking party or eating unhealthy until I get to that bridge, that is what I'm crossing it*.

JAM#6 [JM30, female, 25, student, rural]

In contrast to participants who justified unhealthy behavior in the present, others engaged with food-related videos in an aspirational way, using it to imagine a healthier future version of themselves. Healthier, more abundant eating was often associated with being older, wealthier, and financially stable, suggesting that aspirations were grounded in economic and life-stage realities. In this way, food videos were visual blueprints for imagined futures, allowing young adults to engage positively with ideals that felt out of reach in the present. One UK participant expressed this perspective when discussing a future version of herself.

*When I watch people have so much fresh fruit and vegetables in their fridge, I have this vision of myself when I have more income than I do now, and I think that’s what my life is going to look like. That’s one of the only things I actually really imagined for myself when, I've aged, is that I'm going to get the food products that cost just a little bit more than I have now*.

UK#3 [UK14, female, 23, employed, urban]

Even when young adults tried to adopt healthier diets, current financial realities made these goals seem unattainable. Affordability was a major constraint to translating online influence into offline practice. While food-related videos inspired eating healthy intentions, these were tempered by economic realities that made sustained change difficult for participants in both countries. Some Jamaican participants, as illustrated in the quotes below, reported that the cost of fruits and vegetables was prohibitive and questioned whether the benefits justified the cost.

*I tried buying vegetable once. It was almost more than my grocery bill in total. I will not try it again like that*.

JAM#4 [JM20, female, 22, student, rural]

*Eating healthy is good for you know, but when I look at the prices, brother, it nuh make no sense*.

JAM#1 [JM4, female, 25, employed, rural]

In the United Kingdom, participants similarly described a tension between aspiration and affordability. The cost of variety, freshness, and convenience made healthy choices seem unachievable, especially for students and young adults in the early stages of their careers. Economic limitations reinforced existing habits and constrained the influence of online health messaging. The following quote from a UK participant illustrates this tension.

*I was just thinking that all the good fruits that I like, they're so expensive, like blueberries, strawberries, raspberries, blackberries, so that you're left with like, apples and bananas. I've eaten them [apples and bananas] so much that looking at them makes me feel sick. And so it’s like it’s that struggle of just like… I can't really… I'm a student. I currently spend like £10 on fruit. It’s going to go off … maybe just buy some chicken and make some burgers or just order a burger*.

UK#4 [UK17, female, 25, student, urban]

However, cost did not entirely prevent participants from acting on what they saw in food-related videos. Some adapted, creating homemade versions of popular foods and fakeaways (a “fakeaway” is a homemade version of a takeaway meal; typically a cheaper and customizable alternative to ordering food), to balance aspiration with practicality. These workarounds illustrate how young adults used their resourcefulness to bridge the gap between online influence and their offline reality, participating in digital food trends without exceeding their means. The quote below exemplifies this resourcefulness.

*This is dessert called Trüfrü… [it’s] a strawberry or Raspberry coated and white chocolate or dark chocolate. And it costs about £8 for eight in a pack. So it’s absolutely absurd, but it’s really, really popular and a lot of people having it. And I keep seeing these videos online about how to make them because it’s only like 2-3 pounds to make it for about 20-30. So I literally just made it yesterday [laughs] and yeah, I guess that it does have the influence on me that I have. Yeah, it does make me want to eat unhealthily, I guess*.

UK#6 [UK25, male, 21, student, urban]

Lack of time was another barrier to translating online inspiration into everyday practice. Even when young adults were motivated to prepare healthier meals, they faced competing demands from work, study, and commuting, leaving them with little time for meal preparation. This was a logistical issue that involved an opportunity cost, as participants had to weigh what they must sacrifice to eat well, highlighting that offline decision-making was shaped equally by time-based realities and financial ones. One Jamaican participant articulates this difficulty below.

*The opportunity cost is really a heavy factor. Because even for me, working [I] wake up [at] 6:00 in the morning to get ready for work. Come back after work after six due to traffic. Then I have master’s class from 6:00 to 9:00, which I don’t find most time to cook. Unless I cook from the Sunday before. So really, the opportunity cost is a heavy factor. For me, at least*.

JAM#5 [JM21, male, 25, student, employed, rural]

Not all young adults grappled with a negotiation between online and offline influences. Some participants reported not being influenced to change their diet based on the food-related videos they watched. They described a clear separation between viewing content and altering their eating habits, noting that food-related videos never prompted them to stop eating particular foods or adopt new dietary practices.

*Personally, I haven't come across a video that changed my mind to stop eating anything.*

JAM#1 [JM3, female, 19, employed, urban]

These accounts show that while food-related videos can shape attitudes and intentions, translating influence into action depended on offline realities. Financial constraints, limited time, and competing responsibilities often determined whether young adults implement the ideas or habits they encountered online. Even when motivated, participants weighed the cost and feasibility of changing their routines. Notably, the pattern of digital influence being rejected, accepted, moderated, or adapted in response to daily realities was evident in both countries. However, the way in which it was expressed differed.

### High-Order Theme: Journey of Influence

This overarching theme captures the processes through which young adults experience, interpret, and act on food-related videos. It reflects a journey that begins with exposure, is shaped by algorithms and intentional engagement, progresses through the evaluation of content and is filtered through offline realities, culminating in either a change or continuity of previously held attitudes and behaviors toward food and nutrition. This journey was consistent in both countries. Figure 1 presents a conceptual map of the relationships among the 4 themes that make up this overarching journey.

**Figure 1.**
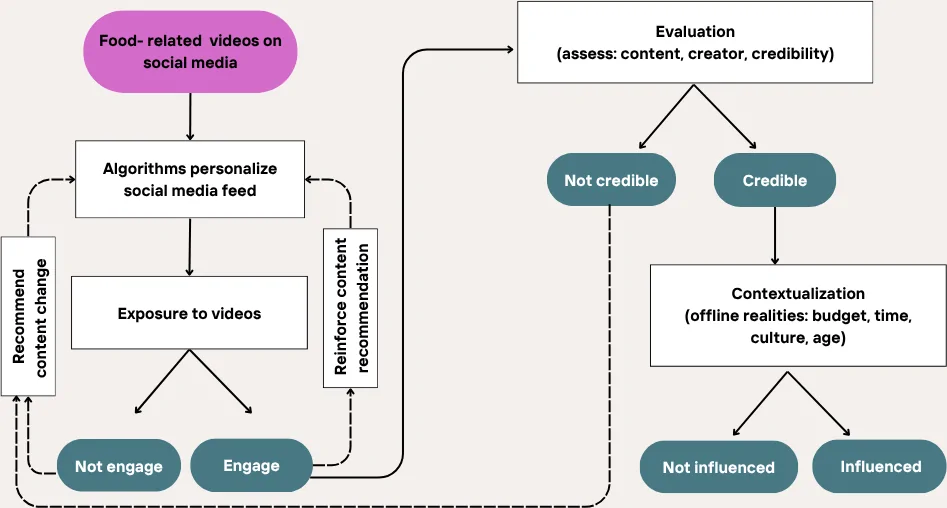
Conceptual map illustrating the process of influence captured in the high-order theme, “a journey of influence,” which integrates the 4 themes, describing how young adults progress from digital exposure to reflection and action. Legend: purple oval=start point; green oval=decision point; rectangle=process; solid arrow=progression from one step to the next; dashed arrow=feedback loop.

Our high-order theme presents three decision points in the journey toward changing attitudes and behaviors: (1) exposure to food-related videos, (2) evaluation of content, and (3) contextualization to offline realities. Each decision point serves as an inflection point for introducing content on healthy eating (exposure), presenting trustworthy, relatable information via credible sources (evaluation), and empowering young adults in their local and personal contexts (contextualization) to make dietary changes.

These decision points are best understood through participants’ own reflections, which illustrate how exposure, evaluation, and contextualization unfold across both country settings. Here, we draw on examples from the United Kingdom and Jamaica that reveal common pathways from online exposure to offline change.

First, a UK participant described her decision to adopt a vegan lifestyle. Her account illustrates how exposure, evaluation, and contextualization converged to inspire behavioral change.

*A few years ago, I think it was like six years now. I watched a video about like a YouTuber that I liked, who was vegan. And it was talking about veganism and then like a couple of months later, that’s what inspired me to become vegan. And so, I feel like that was a direct link and like, it wasn't the main reason, but obviously it was. I watched that video and I'd never really considered it before until I saw that video*.

UK#6 [UK26, nonbinary, 22, employed, urban]

A Jamaican participant reflected a similar journey, in a more subtle way, describing how he began adding avocado to his meals. Although this change was less transformative than becoming vegan, it demonstrates the spectrum of dietary adjustments that can result from the same sequence of exposure, evaluation, and contextualization.

*I started buying a lot of pear [avocado], since recently, after watching all those vegan videos. So, I think it [food-related videos] does. Perhaps not to the greatest degree but as directed to make incremental steps*.

JAM#5 [JM24, male, 23, student, rural]

These examples illustrate how young adults navigate exposure, evaluation, and contextualization to lead to both small and substantial dietary changes.

## Discussion

### Summary

This paper explores the experiences of young adults in Jamaica and the United Kingdom with food-related videos. Four themes were generated, and one high-order theme. The first theme emphasizes how algorithms determine exposure to food-related videos, and how young adults counter this using in-app settings to resist and modify the food-related content they receive. The second theme explores why they continue engaging with food videos, highlighting the roles of relaxation, enjoyment, amusement, and learning. The third theme examines how young adults interrogate and evaluate content, creators, and advertisements using subjective strategies that help them navigate the digital food environment. The fourth theme connects online and offline worlds, showing how cultural context, time, affordability, and life stage determine whether digital content informs real-life attitudes and behaviors. The high-order theme, “a journey of influence,” captures this progression from exposure to potential influence and outlines key decision points where digital and offline realities intersect.

### Comparison With Existing Literature

This study adds to current research by showing how young adults interpret food-related videos and how these interpretations shape whether and how influence occurs. Existing research includes the examination by Drivas et al [8] of the impacts of #WhatIEatInAday TikTok videos on college students’ body image, which included increased body satisfaction, body dissatisfaction, and intention to diet, and Wang et al [12] mixed methods and qualitative [13] explorations of teens’ dietary behaviors after watching food-related videos on TikTok. Our study extends this body of work by examining the interpretive and evaluative processes through which young adults make sense of online food content, offering a deeper understanding of how influence unfolds.

A key finding of this study was the prominence of social media algorithms in shaping participants’ digital food environments. This aligns with work that positions algorithms as powerful social actors that guide and structure online experiences [42,43]. Beer [42] argues that algorithms shape social life through technical filtering and their capacity to determine what becomes visible, legitimate, or desirable. Algorithms, therefore, exert power by normalizing certain types of content and by influencing what users come to see as “worthy” of attention. The work by Swart [43] with young people shows that users experience algorithms as active agents that personalize their feeds, often relying on intuition instead of explicit knowledge to understand how these systems operate. Swart highlights that algorithmic awareness is uneven, as young people recognize the algorithm’s presence, but this does not necessarily lead to any actions. Both researchers describe algorithms as influential forces that shape meaning-making by structuring what people encounter online. Our study builds on these ideas by showing that young adults perceive and respond differently to algorithms. Participants used tactics such as blocking creators, selecting “not interested,” and starting new accounts, illustrating that while algorithms shape exposure, young adults adopt tactics to manage this “social actor” in ways that reflect their cultural contexts and individual priorities.

Our findings on how young adults interpret and respond to food-related videos align with the food-environment framework by Monterrosa et al [44]. They propose that sociocultural factors, such as culture, norms, values, identities, and meaning systems, shape food choices and practices. In our study, cultural identity and norms influenced how participants judged authenticity, evaluated credibility, and engaged with familiar and unfamiliar foods. We extend the framework by Monterrosa et al by demonstrating that food environments are not solely physical or offline; rather, young adults navigate an interface between online and offline worlds, and this intersection is crucial for understanding the influence of digital food environments. Our cross-national comparison develops this finding further. Jamaican participants often drew on a shared national food identity when judging whether food-related videos felt familiar, relevant, or authentic. The work by Sperry [45] on Jamaican food history similarly describes food as a means of creating and reinforcing cultural identity while recognizing cultural traditions within the country. UK participants instead described food through a wider range of ethnic and religious identities rather than through a single “UK culture.” This is consistent with the qualitative research by Ojo et al [46] conducted among ethnically diverse communities in England, which shows that traditional foods, cultural beliefs, and religious practices shape dietary choices and the relevance of nutrition guidance. We do not interpret these patterns as fixed characteristics of either country but see them as proof of how digital influence was interpreted through the cultural reference points that participants considered relevant.

The conceptual map developed in this study also sits alongside frameworks that describe the digital food environment. Granheim et al [47] present a system-level model that visualizes the digital food environment as an extension of the physical food space, structured by platform actors, marketing systems, and technologies. Both their model and our conceptual map recognize the interaction of online and offline influences and the role of human and nonhuman actors (eg, algorithms), but our study provides a user-centered perspective. For example, the framework by Granheim et al maps the architecture of the digital food system while our conceptual map traces how young adults navigate that architecture. It highlights the experiences of young people, specifically how they encounter, interpret, and evaluate content, and how culture, context, and constraints mediate whether digital content translates into meaningful influence. In doing so, our study offers a youth-centered, culturally situated extension of existing models and emphasizes that digital influence is not deterministic.

### Strengths and Limitations

This study has several strengths. The comparative design across Jamaica and the United Kingdom enabled us to examine how young adults in two different contexts interpret and respond to food-related videos, revealing shared patterns in digital food engagement and culturally specific interpretations shaped by local norms and food environments.

Another strength lies in our cocreated and equitable data collection processes. Collaboration with young adults through YPAGs shaped the research agenda and refined study materials, ensuring the topic guide and terminologies reflected youth realities. Piloting the focus groups further strengthened the quality of discussions. To support equitable participation, we offered online and in-person focus groups, increasing accessibility and enabling us to capture a wide range of experiences from young adults with diverse demographic backgrounds.

While we made every effort to minimize the limitations of this study, some remained. Our decision to offer online and in-person focus groups supported inclusive participation, but interaction may have differed between the two formats. As our study was not designed to compare modes of participation, we cannot assess whether format influenced the focus group discussions. Another challenge was the presence of 7 suspected impostor participants. We responded by revising the recruitment strategy, meeting potential participants ahead of the remaining focus groups, and removing the suspected impostor participants’ contributions before analysis. However, focus group discussions are shaped by interactions between participants, and removing their contributions could not remove any influence their presence may have had on other participants’ responses. The disruption also resulted in a smaller UK sample than planned but provided valuable methodological lessons. Additionally, as with all qualitative research, this study’s findings are transferable to similar contexts but not generalizable to all young adults.

### Implications

The conceptual map outlined in Figure 1 presents three decision points in the process of changing attitudes and behaviors: (1) exposure to food-related videos, (2) content evaluation, and (3) contextualization to offline realities. Each point represents an opportunity for further research and public health intervention. However, together, these decision points support a staged and adaptable approach to digital nutrition intervention. The exposure stage suggests a need for research examining how algorithms shape the visibility of food-related videos for different groups of young adults, and how public health campaigns might leverage platform dynamics to increase the visibility of credible content. Possible interventions could support young adults’ agency by helping them shape their feeds and increasing the visibility of credible nutrition content without disregarding the entertainment and social value they derive from food-related videos. The content evaluation stage points to opportunities for interventions that support young adults’ existing vetting strategies, such as helping them recognize credible nutrition information. Potential interventions might enhance digital and nutrition literacy by teaching individuals how to evaluate sources, identify commercial motives, and scrutinize food claims. This effort should focus on guiding young adults to differentiate more reliable checks, such as verifying professional credentials and cross-referencing claims with independent, evidence-based information, from cues that may foster misplaced confidence. Indicators like positive comments, self-declared transparency, perceived authenticity, and AI-generated responses can seem trustworthy but are susceptible to manipulation, commercial bias, or inaccuracies. Consequently, no single signal should be considered as total proof of credibility. Finally, the contextualization stage underscores the importance of designing interventions that acknowledge the realities young adults face, including availability (or lack) of food or cooking resources, affordability of healthy food items, time constraints, life-stage priorities, and the cultural identities that shape their food choices. Public health strategies that incorporate these realities, for instance, by promoting low-cost recipes, culturally relevant guidance, or small changes suited to busy schedules, are more likely to be useful for young adults. Co-designing these components with young adults in each intended setting could help ensure that interventions are engaging, culturally meaningful, and practically achievable. Future research should assess the acceptability, feasibility, and effectiveness of approaches informed by these findings.

Our results provide insight into how risk communication and evaluation operate in digital food environments. Risk communication is defined as “the real-time exchange of information, advice, and opinions between experts or officials and people who face a hazard or threat to their survival, health or economic or social wellbeing” [48]. In nutrition and public health, risk communication may take the form of public health messages and campaigns about food safety, healthy eating habits, and the risks associated with unhealthy diets. In this study, young adults described using informal risk-assessment strategies, such as vetting credibility, identifying red flags, and cross-checking claims, to protect themselves from misleading or exaggerated content online. Understanding these practices can help public health practitioners design messages that young adults are more likely to notice, trust, and act on. Aligning dietary risk communication with the ways young adults actually evaluate information may therefore strengthen the impact of health messages in digital spaces.

Our study provides insights for policies aimed at reducing harms linked to social media. Participants’ feedback highlights the need for increased transparency and user control over algorithm recommendation systems, as well as media and nutrition literacy programs to help users better assess food-related content. They also valued these videos for entertainment, relaxation, and learning, indicating that targeted interventions might be more effective than broad restrictions. Additionally, since online influence was moderated by factors such as food affordability, availability, and cultural norms, social media regulations alone are unlikely to tackle the broader factors influencing food choices.

### Conclusions

Two conclusions can be drawn from our study. First, young adults demonstrate agency in how they engage with food-related content, particularly in evaluating and navigating online influences. The conceptual map illustrates this youth-informed journey, highlighting the active steps young adults take from exposure to response. Second, this agency is exercised within sociocultural and material realities such as limited time, money, and access, which shape how far online influences translate into offline behavior. These factors should be accounted for in dietary interventions and public health campaigns targeting this group.

## Supplementary material

10.2196/89720 Multimedia Appendix 1 The indicative topic guide, screening questionnaire, getting-to-know-participants questionnaire, and stages of data analysis.

10.2196/89720 Checklist 1 COREQ checklist.

## Abbreviations

ASMR: autonomous sensory meridian response
COREQ: Consolidated Criteria for Reporting Qualitative Research
FYP: For You Page
NCD: noncommunicable disease
YPAG: Young People Advisory Group

## References

[1] MacFarlane R (2026). 7 social media trends you need to know in 2026.

[2] Raghavan RS (2022). The rise of video-first marketing.

[3] Fletcher KA, Gbadamosi A (2024). Examining social media live stream’s influence on the consumer decision-making: a thematic analysis. Electron Commer Res.

[4] Guo Y, Shachat J, Walker MJ, Wei L (2021). Viral social media videos can raise pro-social behaviours when an epidemic arises. J Econ Sci Assoc.

[5] (2025). Digital 2025: the state of social media in 2025. DataReportal.

[6] Internet use continues to grow, but universality remains elusive, especially in low-income regions. International Telecommunication Union Facts and Figures 2024.

[7] Does TikTok influence what we eat?. BBC.

[8] Drivas M, Reed OS, Berndt-Goke M (2024). #WhatIEatInADay: the effects of viewing food diary TikTok videos on young adults’ body image and intent to diet. Body Image.

[9] Pryde S, Prichard I (2022). TikTok on the clock but the #fitspo don’t stop: the impact of TikTok fitspiration videos on women’s body image concerns. Body Image.

[10] Luo Y, Maafs-Rodríguez AG, Hatfield DP (2024). The individual-level effects of social media campaigns related to healthy eating, physical activity, and healthy weight: a narrative review. Obes Sci Pract.

[11] Hill BL (2019). The impacts of internet recipe videos on food preferences among young adults: a pilot study [master’s thesis]. https://www.proquest.com/docview/2301477856/abstract/216B2215051C46A5PQ/1.

[12] Wang CH, Sher STH, Salman I, Janek K, Chung CF (2022). IDC ’22: Proceedings of the 21st Annual ACM Interaction Design and Children Conference.

[13] Wang CHA, Sher STH, Chung CF (2024). From viral content to real-life cuisine and beyond: examining teenagers’ interactions with TikTok food videos and the influence on their food practices. Proc ACM Hum-Comput Interact.

[14] Coates AE, Hardman CA, Halford JCG, Christiansen P, Boyland EJ (2019). Food and beverage cues featured in YouTube videos of social media influencers popular with children: an exploratory study. Front Psychol.

[15] Naderer B, Wakolbinger M, Haider S (2024). Influencing children: food cues in YouTube content from child and youth influencers. BMC Public Health.

[16] Smit CR, Buijs L, van Woudenberg TJ, Bevelander KE, Buijzen M (2019). The impact of social media influencers on children’s dietary behaviors. Front Psychol.

[17] Benvenuti M, Arnett JJ, Mazzoni E (2023). Emerging adulthood and media use: latest research and future directions. J Media Psychol.

[18] Lim MSC, Molenaar A, Brennan L, Reid M, McCaffrey T (2022). Young adults’ use of different social media platforms for health information: insights from web-based conversations. J Med Internet Res.

[19] Cowan-Pyle AE, Bailey RL, Gao J (2024). Dietary quality and diet-related factors among emerging adults (18-23 y) in the United States are a cause for concern: National Health and Nutrition Examination Survey 2015-2018. J Nutr.

[20] Poobalan AS, Aucott LS, Clarke A, Smith WCS (2014). Diet behaviour among young people in transition to adulthood (18-25 year olds): a mixed method study. Health Psychol Behav Med.

[21] Matassi M, Boczkowski P (2021). An agenda for comparative social media studies: the value of understanding practices from cross-national, cross-media, and cross-platform perspectives. Int J Commun.

[22] Buzeta C, De Keyzer F, Dens N, De Pelsmacker P (2024). Branded content and motivations for social media use as drivers of brand outcomes on social media: a cross-cultural study. Int J Advert.

[23] Goodrich K, de Mooij M (2014). How ‘social’ are social media? A cross-cultural comparison of online and offline purchase decision influences. J Mark Commun.

[24] United Kingdom-individuals using the Internet. International Telecommunication Union DataHub.

[25] Jamaica - individuals using the Internet. International Telecommunication Union DataHub.

[26] Jamaica: upper-middle income. Global Obesity Observatory.

[27] United Kingdom: High Income. Global Obesity Observatory.

[28] Parks S, Albury C, Renzella J, Kaur A, Rayner M, Scarborough P (2024). Exploring young adults experiences with food-related videos on social media: a focus group study protocol. OSF.

[29] Ayton D, Tsindos T, Berkovic D (2023). Qualitative Research – A Practical Guide for Health and Social Care Researchers and Practitioners.

[30] Olawade DB, Omeni D, Gore MN, Hadi M (2025). Enhancing qualitative research through virtual focus groups and artificial intelligence: a review. Int J Med Inf.

[31] Parks S, Kaur A, Renzella J (2025). Behavioural and cognitive changes in young adults towards food and nutrition after exposure to digital food communication: a mixed-methods systematic review. Int J Behav Nutr Phys Act.

[32] Guest G, Namey E, McKenna K (2017). How many focus groups are enough? Building an evidence base for nonprobability sample sizes. Field Methods.

[33] Malterud K, Siersma VD, Guassora AD (2016). Sample size in qualitative interview studies: guided by information power. Qual Health Res.

[34] Krueger RA, Casey MA (2009). Focus Groups: A Practical Guide for Applied Research.

[35] Lune H, Berg BL (2017). Qualitative Research Methods for the Social Sciences, Global Edition.

[36] Medero K, Abdi H, Ford C, Gollust S (2025). Detecting and preventing imposter participants: methods and recommendations for qualitative researchers. Qual Health Res.

[37] Ridge D, Bullock L, Causer H (2023). “Imposter participants” in online qualitative research, a new and increasing threat to data integrity?. Health Expect.

[38] Braun V, Clarke V, Hayfield N, Davey L, Jenkinson E (2023). Supporting Research in Counselling and Psychotherapy: Qualitative, Quantitative, and Mixed Methods Research.

[39] Gale NK, Heath G, Cameron E, Rashid S, Redwood S (2013). Using the framework method for the analysis of qualitative data in multi-disciplinary health research. BMC Med Res Methodol.

[40] Glaser BG (1965). The constant comparative method of qualitative analysis. Soc Probl.

[41] Davis JL (2017). Curation: a theoretical treatment. Inf Commun Soc.

[42] Beer D (2017). The social power of algorithms. Inf Commun Soc.

[43] Swart J (2021). Experiencing algorithms: how young people understand, feel about, and engage with algorithmic news selection on social media. Soc Media Soc.

[44] Monterrosa EC, Frongillo EA, Drewnowski A, de Pee S, Vandevijvere S (2020). Sociocultural influences on food choices and implications for sustainable healthy diets. Food Nutr Bull.

[45] Sperry AJ (2023). Eating Jamaica: how food is used as a tool to create and reinforce cultural identity. World Hist Connect.

[46] Ojo AS, Nnyanzi LA, Giles EL (2023). “I am not really into the government telling me what I need to eat”: exploring dietary beliefs, knowledge, and practices among ethnically diverse communities in England. BMC Public Health.

[47] Granheim SI, Torheim LE, Terragni L, Thurston M (2025). Conceptualizing the digital food environment: a framework. Urban Plan.

[48] Risk communications. World Health Organization.

