# Supplementary material for: Engagement and Influence of Food-Related Videos on Social Media Within the Dietary Realities of Young Adults in Jamaica and the United Kingdom: Qualitative Cross-National Study
Source: J Med Internet Res. 2026 Oct 19;28:e89720. doi: 10.2196/89720 (PMC13679865; doi:10.2196/89720)
Supplement: Supplementary file 1 [file jmir-v28-e89720-s001.docx]

**Table S1.** Indicative topic guide.

| **TOPICS** | **QUESTIONS & PROMPTS** | **SECTION AIMS** |
| --- | --- | --- |
| Welcome | Welcome and focus group ground rules |  |
| Intro /  Ice breaker (Options) | Let’s learn about each other   - Tell us your name and your favourite season - Tell us your name and a food-related video you saw on social media | Participants practice turn-taking |
| Exposure & Experience | - What type of food-related videos do you often see on social media? - What makes food-related videos appealing/interesting? - Do you watch digital marketing food-related videos from food companies? - Do you prefer digital marketing food-related videos from food companies and paid influencers or food videos that are not associated with a food company? - How do you feel after watching a food-related video? - Do you feel differently if the video is a digital marketing video or one that isn’t associated with a food company? - What happens to you after watching a food-related video? - Does watching videos change what or how you eat? - Does watching food videos change how you think about food or nutrition? | Understand the exposure to food-related videos (digital marketing or organic content) and their experiences |
| Misinformation | - Do you trust the information in food-related videos? - Have you seen any false/misleading videos online? - What are your experiences with false/misleading food-related videos? - Do you think anything can be done to support young people to recognize false/misleading food-related videos? | Learn experiences of misinformation exposure & associated responses. |
| Solutions | - Do you think food-related videos can help people to eat healthily? - How do you think social media (in general) could be used to improve health among young people? | Learn health improvement strategies that young people |
| Closing | Thanks, and the next steps   - Provide details on what to expect next (E.g.: dissemination of vouchers) |  |

**Supplementary Material S1:** Screening Questionnaire

1. Are you aged 18 years to 25 years?
   a. Yes
   b. No
2. Do you currently reside in Jamaica or the UK?
   a. Yes
   b. No
3. Have you resided in Jamaica or the UK for the past 5 years?
   a. Yes
   b. No
4. Do you use any of the following video-based social media such as Tik Tok, Instagram, YouTube, Snapchat, Twitch or any other video-based social media platform?
   a. Yes
   b. No
5. Do you watch food-related videos on social media weekly? E.g.#FoodTikTok
   a. Yes
   b. No

**Section S1.** Getting-To-Know-Participants Questionnaire.

Demographic Information

1. How old are you?
   a. 18 years
   b. 19 years
   c. 20 years
   d. 21 years
   e. 22 years
   f. 23 years
   g. 24 years
   h. 25 years
2. What is your gender?
   a. Male
   b. Female
   c. Non-Binary
   d. Prefer not to say
   e. Other __________
3. Are you currently…

Please select all that apply.

a. A student – Full time
b. A student – Part time
c. Employed – Full time
d. Employed – Part time
e. Self-employed
f. Out of work and looking for work
g. Out of work but not currently looking for work
h. A homemaker
i. Unable to work
j. Other____

4. Where do you live?
a. Jamaica
b. United Kingdom
5. Are you from a…
   a. Rural area (e.g.: village)
   b. Urban area (e.g.: city)
6. Where do you watch food-related videos on social media? [Please select all responses that are relevant]
a. TikTok
b. Instagram
c. YouTube
d. Twitch
e. Snapchat
f. Facebook
g. Other_____________
7. How frequently do you watch…?

| **HOW FREQUENTLY DO YOU WATCH…?** | **MULTIPLE TIMES A DAY** | **ONCE IN A DAY** | **EVERY FEW DAYS** | **WEEKLY** | **MONTHLY** | **SELDOM** |
| --- | --- | --- | --- | --- | --- | --- |
| Restaurant vlogs |  |  |  |  |  |  |
| Recipe videos |  |  |  |  |  |  |
| What-I-Eat-In-A-Day videos |  |  |  |  |  |  |
| Taste tests and reaction videos |  |  |  |  |  |  |
| Grocery haul videos |  |  |  |  |  |  |
| What’s in my fridge videos |  |  |  |  |  |  |
| Health-related/diet videos |  |  |  |  |  |  |
| Food brand/Food product videos |  |  |  |  |  |  |
| Other food influencer videos |  |  |  |  |  |  |
| Private chef videos |  |  |  |  |  |  |

7b. Other types of videos ______________________

Contact Details and Preferences

What email address would you like us to contact you on?

_____________________________

What is your preference for focus groups? (*caveat here – we will try to meet your preferences) – answer all relevant

a. Online (Microsoft Teams)
b. In-person (Oxford, UK; Kingston, Jamaica and Montego Bay, Jamaica)
c. Morning session (10 AM – 11:30 AM)
d. Afternoon session (1 PM – 2:30 PM or 3 PM – 4:30 PM)
e. Evening session (7 PM-8:30 PM)
f. Weekday (Monday - Friday
g. Weekend (Saturday – Sunday)

Do you have any accessibility requirements?

a. Yes
b. No

If yes, please state your accessibility requirements below

___________________________

**Table S2.** Stages of data analysis.

| **No.** | **STAGE AND EXPLANATION** |
| --- | --- |
| **1** | **Familiarisation**: Familiarisation with the data began during the data collection stage. It involved reviewing notes from the focus groups, watching video recordings, listening to audio recordings, and reading transcripts. The task of reviewing and editing transcripts strengthened this process. |
| **2** | **Coding**: We use the term, coding, to describe the process of assigning labels to describe meaning in the data. SP coded data from all focus groups inductively in NVivo 15. Braun & Clarke^1^ served as a guide for coding each segment of meaning. As codes were developed, SP wrote brief definitions for each code and maintained a memo in NVivo to capture reflections about the codes and changes to them throughout the coding process. One focus group from Jamaica and the UK was initially coded and discussed with CA, AK, and PS. Thereafter, a second round of coding was conducted with six focus groups – three from Jamaica and three from the UK. The codes were continuously refined throughout the coding process, and comparisons were made between focus groups within each country and those in the comparator country. SP also used field notes to support analysis, though they were not coded. |
| **3** | **Initial theme generation**: We use the term, themes, to describe patterns, ideas and concepts that repeatedly show up in the data and are meaningful for our research questions. The codes from the first six focus groups were grouped in descriptive categories to create a working analytical framework ^2^, which was shared and discussed with CA, AK, JR, and PS. |
| **4** | **Reviewing and developing themes**: SP’s supervisory team served as critical friends in fortnightly meetings to discuss interpretation of the data, review the codebook, examine the categories developed, and reflect on positionality concerning how SP understood the data. |
| **5** | **Refining, defining and naming themes**: After coding six focus groups, we began the process of reviewing research on the topics and concepts mentioned in the focus groups to refine codes. After coding all twelve focus groups, SP used the sticky notes method ^3^ to visualise merging codes and employed the analytical framework ^2^ to sort codes into updated categories, ultimately developing a final framework matrix. We selected and utilised the ‘one sheet of paper’ (OSOP) method ^4^ to move from descriptive categories to visualise and interpret the themes generated in the focus groups. SP repeated stages four and five after co-authors' feedback on the study's first write-up, leading her to reanalyse data, refine themes, and add a high-order theme. |
| **6** | **Producing the report**: We considered several reporting guidelines but selected the Big Q Qualitative Reporting Guidelines ^5^ to support compilation of the report because it builds on existing reporting guidelines, avoids the pitfalls of methodological inconsistency with qualitative research values, and acknowledges the subjective nature of qualitative research. |

# References

1.	Braun V, Clarke V, Hayfield N, Davey L, Jenkinson E. Doing Reflexive Thematic Analysis. In: Bager-Charleson S, McBeath A, eds. *Supporting Research in Counselling and Psychotherapy : Qualitative, Quantitative, and Mixed Methods Research*. Springer International Publishing; 2022:19-38. doi:10.1007/978-3-031-13942-0_2

2.	Gale NK, Heath G, Cameron E, Rashid S, Redwood S. Using the framework method for the analysis of qualitative data in multi-disciplinary health research. *BMC Med Res Methodol*. 2013;13(1):117. doi:10.1186/1471-2288-13-117

3.	Burgess H, Jongbloed K, Vorobyova A, et al. The “Sticky Notes” Method: Adapting Interpretive Description Methodology for Team-Based Qualitative Analysis in Community-Based Participatory Research. *Qual Health Res*. 2021;31(7):1335-1344. doi:10.1177/10497323211002489

4.	Ziebland S, McPherson A. Making sense of qualitative data analysis: an introduction with illustrations from DIPEx (personal experiences of health and illness). *Med Educ*. 2006;40(5):405-414. doi:10.1111/j.1365-2929.2006.02467.x

5.	Braun V, Clarke V. Reporting guidelines for qualitative research: a values-based approach. *Qual Res Psychol*. 2025;22(2):399-438. doi:10.1080/14780887.2024.2382244
